# Semantic Entropy in Language Comprehension

**DOI:** 10.3390/e21121159

**Published:** 2019-11-27

**Authors:** Noortje J. Venhuizen, Matthew W. Crocker, Harm Brouwer

**Affiliations:** Department of Language Science & Technology, Saarland University, 66123 Saarbrücken, Germany; crocker@coli.uni-saarland.de (M.W.C.); brouwer@coli.uni-saarland.de (H.B.)

**Keywords:** natural language, entropy, neural networks

## Abstract

Language is processed on a more or less word-by-word basis, and the processing difficulty induced by each word is affected by our prior linguistic experience as well as our general knowledge about the world. Surprisal and entropy reduction have been independently proposed as linking theories between word processing difficulty and probabilistic language models. Extant models, however, are typically limited to capturing linguistic experience and hence cannot account for the influence of world knowledge. A recent comprehension model by Venhuizen, Crocker, and Brouwer (2019, *Discourse Processes*) improves upon this situation by instantiating a comprehension-centric metric of surprisal that integrates linguistic experience and world knowledge at the level of interpretation and combines them in determining online expectations. Here, we extend this work by deriving a comprehension-centric metric of entropy reduction from this model. In contrast to previous work, which has found that surprisal and entropy reduction are not easily dissociated, we do find a clear dissociation in our model. While both surprisal and entropy reduction derive from the same cognitive process—the word-by-word updating of the unfolding interpretation—they reflect different aspects of this process: state-by-state expectation (surprisal) versus end-state confirmation (entropy reduction).

## 1. Introduction

Language is processed on a more or less word-by-word basis, and certain words induce more processing effort (as reflected in higher reading times; RTs) than others. Inspired by Shannon’s [1] theory of communication, it has been proposed that the informativity of a word is proportional to the processing effort that it induces. One way to quantify word informativity is using the notion of *surprisal*, which is a metric that quantifies the expectancy of a word [2,3]; the less expected a word is in a given context, the higher its surprisal (also called *self-information*). A second metric for word informativity is the *entropy reduction* induced by a word, which quantifies the extent to which the word decreases the amount of uncertainty about what is being communicated [4]. Surprisal and entropy reduction have been independently proposed as relevant linking hypotheses between probabilistic language models and processing difficulty [5,6,7,8,9,10,11,12,13,14,15]. That is, instantiations of these metrics provide a computational-level explanation (in terms of Marr [16]) of how the probability of a word in a linguistic context (estimated using language models) affects processing difficulty. There exists, however, a range of experimental findings that show that the processing difficulty of individual words is not only affected by their probability as part of the (local) linguistic context but is also affected by the larger discourse and visual context as well as by general knowledge about the world (see, e.g., [17,18,19,20,21,22,23,24,25,26,27,28,29,30,31,32]). Hence, in order to explain these findings in terms of word informativity, the information-theoretic metrics of surprisal and entropy reduction should take into account the probabilistic structure of the world, above and beyond that of the linguistic signal alone. This means that existing instantiations of these information-theoretic metrics, which are generally based on language models, should either be augmented with a probabilistic notion of extra-linguistic knowledge or be redefined in terms of the underlying cognitive processes.

In this paper, we take the latter approach by building upon previous work by Venhuizen et al. [33] (henceforth, VCB), who put forward a model of language comprehension in which surprisal estimates are derived from the probabilistic, distributed meaning representations that the model constructs on a word-by-word basis. By systematically manipulating the model’s linguistic experience (the linguistic input history of the model) and world knowledge (the probabilistic knowledge captured within the representations), VCB show that, like human comprehenders, the model’s comprehension-centric surprisal estimates are sensitive to both of these information sources. Since surprisal in this model directly derives from the process of incremental linguistic comprehension, the model offers an explanation at Marr’s representational and algorithmic level of how linguistic experience and world knowledge can affect processing difficulty as quantified by surprisal. Given that entropy reduction has been argued to be a relevant predictor of processing difficulty independent of surprisal [15], we here extend these results by deriving a comprehension-centric metric of entropy from the meaning representations that the model constructs. Whereas previous instantiations of entropy in language are defined over linguistic structures (e.g., Probabilistic Context-Free Grammar, PCFG, states [4,14], parts-of-speech [8], or individual words [15]), we here define entropy as the amount of uncertainty relative to the state of affairs of the world. That is, the entropy reduction of a word $w_{t}$ quantifies how much uncertainty regarding the current state of affairs is taken away by processing word $w_{t}$. Empirical support for such an approach comes from a recent study of situated language comprehension, which manipulated only the visual context, thus keeping (linguistic) surprisal constant [34]. Words that reduce referential entropy to a greater extent—with respect to a visual context—led to increased processing effort for otherwise identical utterances.

We investigate whether the comprehension-centric notions of surprisal and entropy reduction make differential predictions within the model and how these metrics relate to the underlying cognitive process of comprehension. Based on the results, we conclude that surprisal and entropy reduction derive from a single cognitive process—comprehension as navigation through meaning space—and that they reflect different aspects of this process: state-by-state expectation (surprisal) versus end-state confirmation (entropy reduction). Critically, while previous language model-based instantiations have found that surprisal and entropy reduction are not easily dissociated [15], the comprehension-centric perspective on word informativity predicts that surprisal and entropy reduction differentially reflect effects of linguistic experience and world knowledge during online comprehension.

In what follows, we first introduce the probabilistic, distributed meaning representations used by VCB [33], from a novel, formal semantic perspective (cf. [35]) (Section 2.1). Next, we describe the comprehension model (Section 2.2.1) as well as how processing in this model gives rise to a comprehension-centric notion of surprisal (Section 2.2.2). From here, a comprehension-centric notion of entropy is derived (Section 2.3). The remainder of the paper, then, explores how and why comprehension-centric entropy reduction differs from comprehension-centric surprisal (Section 3). Finally, we discuss the implications of our findings and outline directions for further study (Section 4).

## 2. Comprehension-Centric Surprisal and Entropy

VCB [33] present a computational model of language comprehension that explicates how world knowledge and linguistic experience are integrated at the level of interpretation and combine in determining online expectations. To this end, they present a neural network model that constructs a representation of utterance meaning on an incremental, word-by-word basis. It is shown that word surprisal naturally derives from the incremental construction of these meaning representations, and that it is affected by both linguistic experience (the linguistic input history of the model) and world knowledge (the probabilistic knowledge captured within the representations). Here, we will show that in addition to this comprehension-centric notion of surprisal, the meaning representations also allow for the definition of a comprehension-centric notion of entropy.

### 2.1. Meaning in a Distributional Formal Meaning Space

The notion of surprisal presented in [33] exploits the rich, probabilistic meaning representations that are constructed by the comprehension model on a word-by-word basis. These representations, which are based on the Distributed Situation-state Space framework [36,37], are argued to instantiate situation models that allow for world knowledge-driven inference. Following [35], we here reconceptualize this approach in terms of model-theoretic semantics, thereby emphasizing the generalizability of the framework.

**Figure 1:**
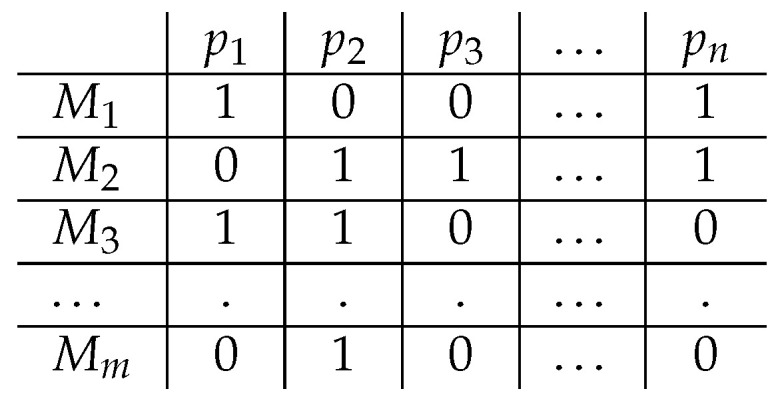
Example of a meaning space $S_{M\times P}$, where $M=\{M_{1},\ldots ,M_{m}\}$ defines the set of models and $P=\{p_{1},\ldots ,p_{n}\}$ the set of propositions. Rows represent models as combinations of propositions and columns represent meaning vectors that derive from this space, such that: ${\vec{v}}_{i}\left(p_{j}\right)=1\mathrm{iff}M_{i}⊧p_{j}$.

Based on a set of propositions P, and a set of models formal models M (which can be defined as combinations of the propositions in P), we can define a meaning space: $S_{M\times P}$ (see Figure 1). Importantly, the set of models M is assumed to reflect the state of the world truth-conditionally and probabilistically (i.e., reflecting the probabilistic structure of the world). The meaning of a proposition p∈P is defined as the vector $\vec{v}\left(p\right)$ that, for each M∈M, assigns a 1 iff *M* satisfies *p* (M⊧p) and a 0 otherwise. The resulting meaning vector captures the truth conditions of individual propositions indirectly by identifying the models that satisfy the proposition. Because the meaning vectors of all propositions are defined with respect to the same set of models, the distributional meaning of any p∈P is defined in relation to all other $p^{\prime }\in P$; that is, propositions that have related meanings will be true in many of the same models and hence have similar meaning vectors.

Given well-defined sets of models M and propositions P (i.e., P fully describes the set of propositions that can be captured in M), the resulting vector space $S_{M\times P}$ offers distributed representations that are compositional and probabilistic. To start with the former, the meaning space $S_{M\times P}$ not only allows for deriving the meaning vectors of individual propositions in P but also combinations thereof. That is, given a definition of negation and conjunction over meaning vectors, the meaning of any logical combination of propositions in the semantic space can be defined. The meaning vector $\vec{v}\left(p\right)$ of a proposition p∈P defines its truth values relative to M, which means that we can define its negation $\vec{v}\left(\neg p\right)$ as the vector that assigns 0 to all M∈M such that *p* is satisfied in *M* and 1 otherwise:

(1) $${\vec{v}}_{i}\left(\neg p\right)=1\;\mathrm{iff}\;M_{i}⊭p\;\mathrm{for}\;1\leq i\leq \left|M\right|.$$

The meaning of the conjunction p∧q, given p,q∈P, then, is defined as the vector $\vec{v}\left(p\wedge q\right)$ that assigns 1 to all M∈M such that *M* satisfies both *p* and *q* and 0 otherwise:

(2) $${\vec{v}}_{i}\left(p\wedge q\right)=1\;\mathrm{iff}\;M_{i}⊧p\;\mathrm{and}\;M_{i}⊧q\;\mathrm{for}\;1\leq i\leq \left|M\right|.$$

The probabilistic nature of the meaning space $S_{M\times P}$ derives from the fact that the meaning vectors for individual propositions in $S_{M\times P}$ inherently encode their probability. Given a set of models M that reflects the probabilistic nature of the world, the probability of any formula φ can be defined by the number of models that satisfy φ, divided by the total number of models:

(3) P(φ)=|{M∈M|M⊧φ}|/|M|.

Thus, (logical combinations of) propositions that are true in a large set of models will obtain a high probability and vice versa. Given that this directly allows for the definition of the conjunctive probability of two formulas, we can also define the conditional probability of any formula ψ given φ:

(4) P(ψ|φ)=P(φ∧ψ)/P(φ).

In order to obtain sensible probability estimations about propositional co-occurrence in $S_{M\times P}$, the set of models M needs to reflect the probabilistic structure of the actual world regarding the truth-conditions and co-occurrence of each proposition p∈P. Arriving at such a set of models M is a non-trivial exercise. One possible strategy would be to deduce the meaning space from annotated corpora or knowledge bases with world knowledge-driven inferences (e.g., [38]), or from crowd-sourced human data on propositional co-occurrence (e.g., [39]). However, in order to empirically evaluate how the information-theoretic notion of entropy (reduction) is affected by the structure of the world, the co-occurrence between propositions needs to be defined in a controlled manner. Therefore, the meaning representations used here (following VCB [33]) are induced from a high-level description of the structure of the world, using an incremental, inference-driven construction procedure [35].

### 2.2. A Model of Surprisal Beyond the Words Given

#### 2.2.1. The Comprehension Model

**Figure 2:**
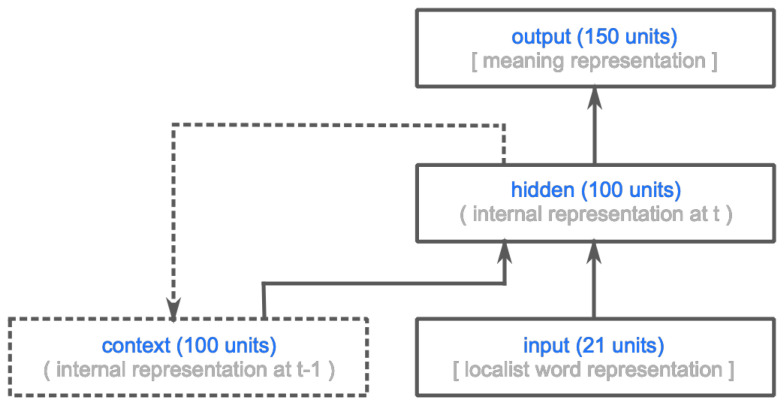
Graphic depiction of the simple recurrent neural network from [33]. Boxes represent groups of artificial neurons, and solid arrows between boxes represent full projections between the neurons in a projecting and a receiving group. The dashed lines indicate that the context layer receives a copy of the activation pattern at the hidden layer at the previous time-step. See text for details.

The model presented by VCB [33] is a simple recurrent neural network (SRN) [40] consisting of three groups of artificial logistic dot-product neurons: an input layer (21 units), hidden layer (100 units), and output layer (150 units) (see Figure 2). Time in the model is discrete, and at each processing time-step *t*, activation flows from the input through the hidden layer to the output layer. In addition to the activation pattern at the input layer, the hidden layer also receives its own activation pattern at time-step t−1 as input (effectuated through an additional context layer, which receives a copy of the activation pattern at the hidden layer prior to feed-forward propagation). The hidden and the output layers both receive input from a bias unit (omitted in Figure 2). The model was trained using bounded gradient descent [41] to map sequences of localist word representations constituting the words of a sentence onto a meaning vector from $S_{M\times P}$, representing the meaning of that sentence.

The sentences on which the model is trained describe situations in a world that is defined in terms of three persons (p∈{beth,dave,thom}), two places (x∈{cinema,restaurant}), two types of food (f∈{dinner,popcorn}), and three drinks (d∈{champagne,cola,water}), which can be combined using the following seven predicates: *enter(p,x)*, *ask_menu(p)*, *order(p,f/d)*, *eat(p,f)*, *drink(p,d)*, *pay(p)*, and *leave(p)*. The resulting set of propositions P (|P|=45) fully describes the world. A meaning space was constructed from these atomic propositions by sampling a set of 10K models M, while taking into account world knowledge in terms of hard and probabilistic constraints on propositional co-occurrence; for instance, a person can only enter a single place (hard), ordering water is more common than ordering champagne (probabilistic), and eating popcorn is more likely in the cinema than in the restaurant (probabilistic) (see [33] for details). In order to employ meaning vectors derived from this meaning space in the SRN, a subset $M^{\prime }$ consisting of 150 models was algorithmically selected from M, such that $M^{\prime }$ adequately reflected the structure of the world ([33], Appendix B). Situations in the world were described using sentences from a language consisting of 21 words. The grammar described in [33] generates a total of 117 different (active) sentences, consisting of simple noun phrase-verb phrase (NP VP) sentences and coordinated (NP VP and VP) sentences. Sentence-initial NPs identify persons, and VPs directly map onto the aforementioned propositions. The semantics assigned to the sentences were meaning vectors from $S_{M\times P}$ reflecting propositional (simple sentences) or conjunctive meanings (coordinated sentences). In order to induce differential linguistic experience in the model, some of these sentences were encountered more often than others during training; in particular, the sentences “*NP${}_{person}$ ordered dinner/champagne*” occurred nine times more often than “*NP${}_{person}$ ordered popcorn/water*” (whereas the frequency of the different NPs was held constant throughout the training set, see [33] for details). The resulting training set consisted of 237 sentences, which the model encountered 5000 times during training (see [33] for details on other training parameters).

After training, the model successfully learned to map sequences of word representations (representing sentences) onto meaning vectors from $S_{M\times P}$ that describe the semantics of these sentences. Since the aim is to investigate how information-theoretic metrics can be derived from the processing behavior of the model, the effects need to be tightly controlled, which is why the model is not tested using a separate set of unseen test sentences (note, however, that other models employing similar meaning representations have shown generalization to unseen sentences and semantics, in both comprehension [37] and production [42]). Instead, the performance of the model was evaluated using a comprehension score *comprehension(a,b)* [37] that indicates how well meaning vector *a* is understood to be the case from meaning vector *b*, resulting in a score that ranges from −1 (perfectly understood not to be the case) to +1 (perfectly understood to be the case). The average comprehension score of the intended target given the model’s output vector over the entire training set was 0.89, which means that after processing a sentence, the model almost perfectly infers the intended meaning of the sentence. This shows that, due to the structured nature of the meaning representations, the (rather simple) SRN architecture suffices to obtain the desired comprehension behavior. It should be noted, however, that the meaning representations could also be employed in a more cognitively plausible architecture, in order to gain more insight into the cognitive processes underlying incremental language comprehension, for instance, by linking model behavior to electrophysiological correlates [43].

#### 2.2.2. A Comprehension-Centric Notion of Surprisal

**Figure 3:**
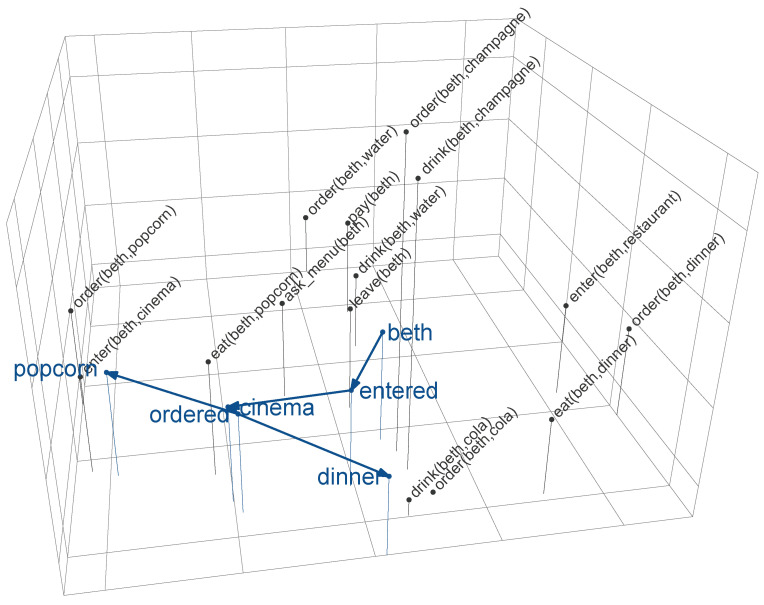
Three-dimensional visualization of the meaning space (by means of multidimensional scaling) for a subset of the atomic propositions (those pertaining to *beth*). Coloured points and arrows show the word-by-word navigational trajectory of the model from [33] for the sentence *beth entered the cinema and ordered [popcorn/dinner]* (function words are omitted; see text for details).

On the basis of its linguistic input, the comprehension model incrementally constructs a meaning vector at its output layer that captures the meaning of the sentence so far; in other words, the model effectively navigates the meaning space $S_{M\times P}$ on a word-by-word basis. That is, each incoming word $w_{t}$ induces a transition from a point in meaning space ${\vec{v}}_{t-1}$ to the next ${\vec{v}}_{t}$. Figure 3 provides a visualization of this navigation process. This figure is a three-dimensional representation of the 150-dimensional meaning space (for a subset of the atomic propositions), derived using multidimensional scaling (MDS). The grey points in this space correspond to propositional meaning vectors. As this figure illustrates, meaning in $S_{M\times P}$ is defined in terms of co-occurrence; propositions that co-occur frequently in M (e.g., *order(beth,cola)*, and *drink(beth,cola)*) are positioned close to each other in space. Note that multidimensional scaling from 150 into three dimensions necessarily results in a significant loss of information; therefore, distances between points in the meaning space shown in Figure 3 should be interpreted with care. The coloured points show the model’s word-by-word output for the sentences “*beth entered the cinema and ordered [popcorn/dinner]*” (as the function words “*the*” and “*and*” trigger minimal transitions in meaning space, they are left out in Figure 3 to enhance readability). The navigational trajectory (indicated by the arrows) illustrates how the model assigns intermediate points in meaning space to each (sub-sentential) sequence of words. For instance, at the word “*beth*”, the model navigates to a point in meaning space that is in between the meanings of the propositions pertaining to *beth*. The prior probability of propositions in $S_{M\times P}$ (“world knowledge”), as well as the sentences on which the model was trained (“linguistic experience”) together determine the model’s trajectory through meaning space. For instance, while the model was exposed to the sentences “*beth entered the restaurant and ordered popcorn*” and “*beth entered the restaurant and ordered dinner*” equally often, the meaning vector at the word “*ordered*” is closer to *order(beth,popcorn)* (cos(θ)=0.70) than to *order(beth,dinner)* (cos(θ)=0.16), because the former is more probable in the model’s knowledge of the world (see [33] for details).

Based on the view of comprehension as meaning space navigation, VCB [33] define surprisal in terms of the points in meaning space that the model incrementally constructs. As a result, surprisal in the model essentially reflects the distance of transitions in meaning space: in case the meaning vector after processing word $w_{t}$ (i.e., ${\vec{v}}_{t}$) is close to the previous point in meaning space ${\vec{v}}_{t-1}$, the transition induced by word $w_{t}$ is small, indicating that this word is unsurprising. If, on the other hand, ${\vec{v}}_{t}$ is far away from ${\vec{v}}_{t-1}$, the transition induced by word $w_{t}$ is big, and thus, this word is highly surprising. Because of the probabilistic nature of the meaning representations that are derived from $S_{M\times P}$, the conditional probability $P({\vec{v}}_{t}|{\vec{v}}_{t-1})$ can be calculated directly from the meaning vectors (see Equation (4)). This, then, results in the following definition of surprisal:

(5) $$S\left(w_{t}\right)=-\log P\left({\vec{v}}_{t}|{\vec{v}}_{t-1}\right).$$

That is, the surprisal induced by word $w_{t}$ is inversely proportional to the conditional probability of the meaning vector constructed after processing word $w_{t}$, given the meaning vector constructed after processing words $w_{1},\ldots ,w_{t-1}$. VCB [33] show that this comprehension-centric notion of surprisal is sensitive to both the *world knowledge* represented in the meaning representations as well as to the *linguistic experience* of the model. World knowledge derives from the probabilistic structure of the meaning space, as determined by the truth-conditional and probabilistic co-occurrences between propositions in M. As a result, (sub-)propositional meaning vectors that are likely to co-occur in the world will be close to each other in meaning space. Hence, the surprisal of word $w_{t}$ will be affected by how likely its resultant meaning vector ${\vec{v}}_{t}$ is to co-occur with the previous meaning vector ${\vec{v}}_{t-1}$ in M. The linguistic experience of the model, in turn, is determined by frequency differences within the set of training items. When certain sentence-final meaning vectors occur more frequently in the training data, this will affect the word-by-word navigation of the model through meaning space; that is, the meaning vector constructed at word $w_{t-1}$ will be closer to the more frequent sentence-final meanings than to the less frequent ones. As a result, surprisal of word $w_{t}$ will be lower if $w_{t}$ moves the model towards a point in space that is closer to a more frequent sentence-final meaning vector. Crucially, since world knowledge and linguistic experience in the model derive from different probability distributions (i.e., over models versus training items), they need not be in unison. VCB [33] show that their notion of surprisal reflects a weighted average predictability derived from both of these sources.

### 2.3. Deriving a Comprehension-Centric Notion of Entropy

Entropy is a metric that quantifies the amount of uncertainty in a given state. In the context of language processing, entropy reduction defines the extent to which a word decreases the amount of uncertainty about what is being communicated, which is hypothesized to affect cognitive processing [4,14,44]. In terms of the model presented above, language comprehension can be viewed as navigating the meaning space $S_{M\times P}$ on a word-by-word basis (see again Figure 3). At each point in time *t*, the model finds itself at a point in meaning space, defined by the meaning vector ${\vec{v}}_{t}$, that reflects the meaning of words $w_{1},\ldots ,w_{t}$ (as derived from the linguistic experience and world knowledge available to the model). This navigational process effectively aims to recover which combinations of propositions satisfy the meaning at time *t*. That is, at each point in space the model tries to determine the current state of affairs in terms of the propositions in P (i.e., each p∈P is either true or false). In other words, the meaning vector ${\vec{v}}_{t}$ inherently reflects uncertainty about which fully specified state of affairs corresponds to the current point in space. The notion of entropy can be used to quantify this uncertainty.

Given |P|=n, there are $2^{n}$ fully specified states of affairs. In order to calculate entropy, we need a probability distribution over this entire set. This will, however, quickly become infeasible (in the current model, |P|=45, resulting in $2^{45}>10^{13}$ probabilities) [45]. Critically, however, not all combinations of propositions are licensed by world knowledge; only those states of affairs that correspond to one of the models that constitutes the meaning space will obtain a probability P>0 (since all other combinations will yield the zero vector $\vec{0}$). That is, the models in M themselves represent fully specified states of affairs, which, like any other combination of propositions, can be represented as a meaning vector ${\vec{v}}_{M_{i}}$ (e.g., ${\vec{v}}_{M_{1}}=\vec{v}\left(p_{1}\wedge \neg p_{2}\wedge \ldots \wedge p_{n}\right)$; see Figure 1), which will have a 1 for exactly that unit that corresponds to model $M_{i}$. By definition these model vectors inherently carry a probability, which can be used to define entropy. To this end, we define a probability distribution over the set of meaning vectors that identify unique models in M, i.e., $V_{M}=\left\{{\vec{v}}_{M}\;\right|\;{\vec{v}}_{M}\left(i\right)=1\mathrm{iff}M_{i}=M$ and *M* is a unique model in M}. The probabilities of the unique models in $V_{M}$ form a proper probability distribution since they are by definition mutually exclusive $(P({\vec{v}}_{1}\wedge {\vec{v}}_{2})=0$ for each ${\vec{v}}_{1},{\vec{v}}_{2}\in V_{M}$ such that ${\vec{v}}_{1}\neq {\vec{v}}_{2})$, and their probabilities sum to 1 since $V_{M}$ covers the entire meaning space: ${⋁}_{\vec{v}\in V_{M}}=\vec{1}$. At time step *t*, entropy can then be defined as follows:

(6) $$H\left(t\right)=-\sum_{{\vec{v}}_{M}\in V_{M}}P\left({\vec{v}}_{M}|{\vec{v}}_{t}\right)\log P\left({\vec{v}}_{M}|{\vec{v}}_{t}\right).$$

Following this definition, entropy will be zero if the current meaning vector ${\vec{v}}_{t}$ singles out a unique model. If, on the other hand, all models are equally likely at *t* (i.e., the probability distribution over all possible models is uniform), entropy will be maximal with respect to *t*.

In the psycholinguistic literature, entropy has been linked to processing difficulty via the entropy reduction hypothesis (ERH), which states that the reduction of entropy “is positively related to human sentence processing difficulty” ([4], p. 650). The entropy reduction between two states, as triggered by word $w_{t}$, is defined as the difference between the entropy at state t−1 and the entropy at state *t*:

(7) $$\Delta H\left(w_{t}\right)=H\left(t-1\right)-H\left(t\right).$$

In terms of the comprehension-centric notion of entropy, this means that an increase in processing effort is predicted for words that more greatly reduce uncertainty about fully specified states of affairs. Crucially, however, the difference in entropy between time step t−1 and *t* is not necessarily positive; that is, as the model navigates the meaning space on a word-by-word basis, individual words may in principle result in either an increase or a decrease in the uncertainty about which state of affairs is being communicated. While there is no negative entropy reduction in the current model due to the structure of the training data in which the coordinated sentences describe increasingly specific states of affairs, training the model to achieve broader empirical coverage may lead it to exhibit behavior in which it finds itself in a state of relative certainty about the communicated state of affairs, which is then challenged by additional input that moves the model toward a state of increased uncertainty (note that any slightly negative entropy reduction values shown in the plots below result from noise due to the processing behavior of the model). Hence, just as a decrease in entropy reflects the transition from a state of uncertainty to a state of greater certainty, an increase in entropy reflects the transition from a state of certainty to a state of uncertainty. As a result, both positive and negative changes in uncertainty are predicted to increase processing effort. Thus, in contrast to the—syntactically defined—ERH from [4], according to which entropy reduction (and hence, processing effort) is zero in case the current state is more uncertain than the previous state, the comprehension-centric perspective on entropy predicts that both a reduction and an increase in entropy result in an increase in processing effort. That is, the processing difficulty indexed by entropy reduction is a direct reflection of the absolute degree of change in (un)certainty ($\left|\Delta H(w_{t})\right|$) about the communicated state of affairs, as induced by word $w_{t}$: the larger the change in (un)certainty between state ${\vec{v}}_{t-1}$ prior to processing word $w_{t}$ and state ${\vec{v}}_{t}$ after processing $w_{t}$, the higher the processing difficulty.

In what follows, we will investigate how these comprehension-centric notions of entropy and entropy reduction behave in the online comprehension model from [33] and how they relate to the notion of surprisal described in Section 2.2.2.

## 3. Entropy Reduction in Online Comprehension

### 3.1. Comprehension-Centric Entropy Reduction versus Surprisal

Surprisal and entropy reduction have been independently proposed as a linking theory between probabilistic language models and human processing difficulty in online word-by-word comprehension [2,3,4]. Moreover, it has been shown that these information theoretic metrics also independently account for variability in word processing difficulty [15]. A first step, therefore, is to examine the degree to which the predictions of the comprehension-centric instantiations of surprisal and entropy reduction align in the model.

**Figure 4:**
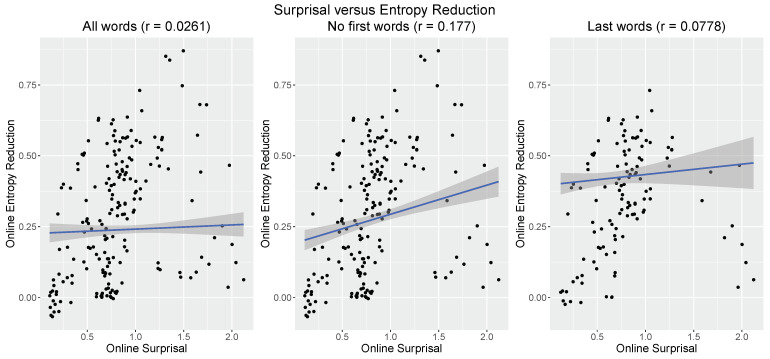
Comparison between online entropy reduction and online surprisal estimates. The scatter plots show the correlation between the surprisal and entropy reduction estimates for all words (**left**), all but the first words (**middle**), and for the last words only (**right**). The solid blue lines depict the corresponding linear regressions with their 95% confidence intervals. The Pearson correlation efficient (*r*) is shown at the top of each plot.

Figure 4 (left) plots the online surprisal estimates for each training sentence of the VCB model [33] against the corresponding online entropy reduction estimates (we here use the term “online” in order to differentiate the model-derived surprisal and entropy reduction metrics from the “offline” metrics derived from the model’s training data; see Section 3.2 below). Overall, there is no significant relationship between the two metrics (r=0.0261, p=0.408). However, given that sentence-initial words minimally reduce uncertainty about sentence-final meaning (all sentences start with a proper name, and all models in M satisfy at least one proposition concerning each person), they induce a rather uniform surprisal (mean = 1.24, sd = 0.01) and entropy reduction (mean = 0.107, sd = 0.01) profile, which may cloud the relationship between these metrics. To account for this, Figure 4 (middle) shows the estimates for all but the sentence-initial word. This now reveals a significant relationship between surprisal and entropy reduction (r=0.177, p<0.01), albeit a weak one ($R^{2}=0.0315$), leaving the majority of variance unaccounted for. Finally, as the last word of an utterance maximally disambiguates (or confirms anticipated) utterance meaning, it is also of interest to look at these separately. Figure 4 (right) plots the estimates for all sentence-final words. At this position, there is no significant relationship between surprisal and entropy reduction (r=0.0778, p=0.233).

In summary, when we ignore the rather uniform surprisal and entropy reduction profiles at the sentence-initial words, we observe a weak positive correlation between the two metrics. This relationship, which does not appear to be driven by disambiguation or confirmation at sentence-final words, explains about 3% of the variance and hence leaves the majority of variability unaccounted for. This raises the question of where and how the comprehension-centric instantiations of the metrics diverge. VCB [33] explored the online surprisal metric by investigating its sensitivity to different degrees of linguistic experience and probabilistic world knowledge. Hence, one way forward is to examine the sensitivity of online entropy reduction under these constellations and to identify where and how it differs from online surprisal.

### 3.2. Effects of Linguistic Experience versus World Knowledge

The comprehension model of VCB [33] maps sentences onto their their corresponding probabilistic meaning vectors on an incremental, word-by-word basis. Crucially, the model is exposed to certain sentence-semantic pairs more frequently than others during training, thereby shaping its linguistic experience. In addition, as each meaning vector inherently carries its own probability in the meaning space, certain sentences can map onto meanings that are more likely than others, which provides the model with world knowledge. These individual sources of knowledge, which influence the behavior of the model, can be independently quantified in the training data using surprisal.

The linguistic experience that the model is exposed to can be quantified using the offline *linguistic* surprisal, which is straightforwardly estimated from the sentences that the model is trained on [2,3]:

(8) $$\begin{array}{cc} S_{ling}\left(w_{t}\right) & =-\log P(w_{t}\;|\;w_{1,\ldots ,t-1}). \end{array}$$

If a word $w_{t}$ frequently occurs after the prefix $w_{1},\ldots ,w_{t-1}$, its conditional probability will be high and its linguistic surprisal low (and vice versa). Crucially, this linguistic surprisal metric is not influenced by the world knowledge contained within meaning vectors; it solely derives from the distribution of word sequences in the set of training sentences.

World knowledge, in turn, can be quantified using offline *situation* surprisal, which is derived from the meaning vectors corresponding to the training sentences, rather than the sentences themselves. That is, given a sequence of words $w_{1},\ldots ,w_{t}$, a situation vector $\mathrm{sit}(w_{1,\ldots ,t})$ can be derived by taking the disjunction of the semantics of all sentences that are consistent with this prefix. For instance, the situation vector of the prefix `Dave drank’ is defined as $\mathrm{sit}\left(\mathrm{Dave}\;\mathrm{drank}\right)=\vec{v}\left(drink\left(dave,water\right)\vee drink\left(dave,cola\right)\vee drink\left(dave,champagne\right)\right)$, the disjunction of all meaning vectors consistent with the word sequence “Dave drank”. The offline situation surprisal induced by a next word is then defined as follows:

(9) $$S_{sit}\left(w_{t}\right)=-\log P\left(\mathrm{sit}\left(w_{1,\ldots ,t}\right)\;|\;\mathrm{sit}\left(w_{1,\ldots ,t-1}\right)\right).$$

If an incoming word $w_{t}$ leads to a situation vector that is highly likely given the situation vector for the disjunctive semantics consistent with the words $w_{1},\ldots ,w_{t-1}$, its conditional probability—which is estimated through its conditional belief—will be high and its situation surprisal low and vice versa. This offline situation surprisal metric is independent of linguistic experience; it is only sensitive to probabilistic world knowledge encoded within the meaning space.

By differentially manipulating linguistic experience and world knowledge, VCB [33] investigate the behavior of their comprehension-centric, online surprisal metric under three constellations:

1. Manipulation of linguistic experience only: the model is presented with sentences that differ in terms of their occurrence frequency in the training data (i.e., differential linguistic surprisal) but that keep the meaning vector probabilities constant (i.e., equal situation surprisal).
2. Manipulation of world knowledge only: the model is presented with sentences that occur equally frequently in the training data (i.e., equal linguistic surprisal) but differ with respect to their probabilities within the meaning space (i.e., differential situation surprisal).
3. Manipulation of both linguistic experience and world knowledge: to investigate the interplay between linguistic experience and world knowledge, the model is presented with sentences in which the linguistic experience and world knowledge are in conflict with each other (i.e., linguistic experience dictates an increase in linguistic surprisal whereas world knowledge dictates a decrease in situation surprisal or vice versa).

**Figure 5:**
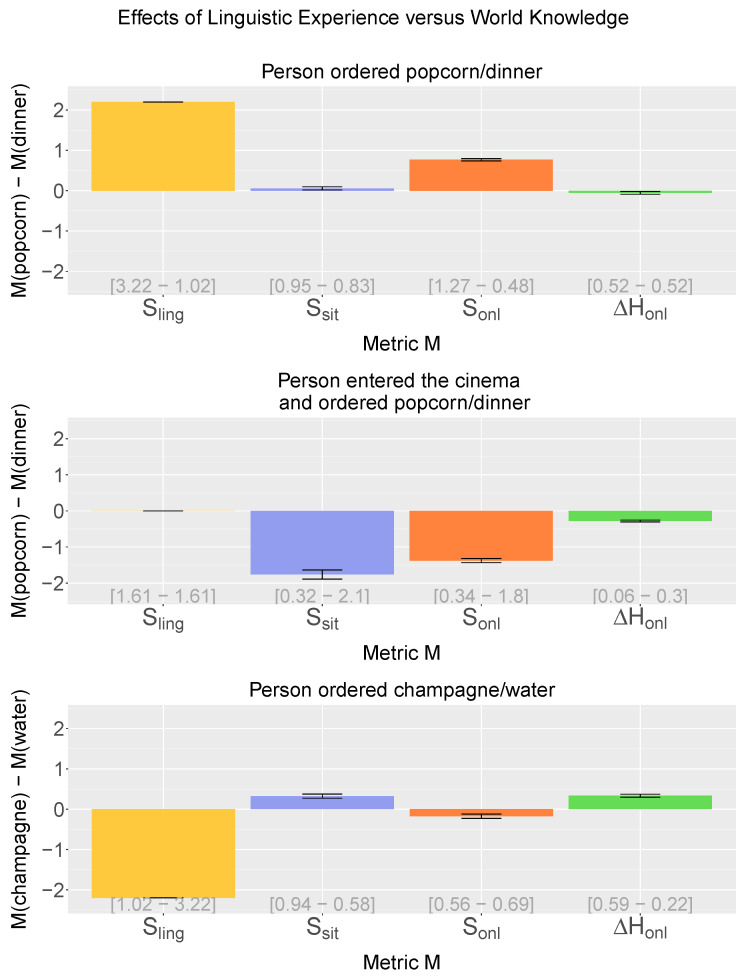
Effects of linguistic experience (LE) versus world knowledge (WK) on linguistic surprisal (S${}_{\mathrm{ling}}$), situation surprisal (S${}_{\mathrm{sit}}$), online surprisal (S${}_{\mathrm{onl}}$), and online entropy reduction (ΔH${}_{\mathrm{onl}}$). Bars represent differences between two target words. **Top**: Effects of LE for the contrast “*NP${}_{person}$ ordered popcorn* [T]/*dinner* [C]”. **Middle**: Effects of WK for “*NP${}_{person}$ entered the cinema and ordered popcorn* [T]/*dinner* [C]”. **Bottom**: Interplay between LE and WK for the contrast “*NP${}_{person}$ ordered champagne* [T]/*water* [C]”. Error bars show standard errors (n = 3). Individual means are shown in brackets (T-C).

Here, we compare the comprehension-centric notion of online entropy reduction (ΔH${}_{\mathrm{onl}}$; see Equations (6) and (7)) to online surprisal (S${}_{\mathrm{onl}}$; see Equation (5)) under these three constellations, which are constructed by manipulating offline linguistic surprisal (S${}_{\mathrm{ling}}$; see Equation (8)), reflecting the linguistic experience of the model, and offline situation surprisal (S${}_{\mathrm{sit}}$; see Equation (9)), reflecting the world knowledge available to the model. Figure 5 shows the difference in surprisal/entropy reduction for these manipulations.

When only linguistic experience is manipulated (the sentence “*NP${}_{person}$ ordered dinner*” is more frequent than “*NP${}_{person}$ ordered popcorn*”) and world knowledge is held constant (P(order(dinner))=P(order(popcorn)) in the meaning space), online surprisal (S${}_{\mathrm{onl}}$) pairs with offline linguistic surprisal (S${}_{\mathrm{ling}}$) in that “popcorn” is more effortful than “dinner”. Online entropy reduction (ΔH${}_{\mathrm{onl}}$), in turn, like offline situation surprisal (S${}_{\mathrm{sit}}$), shows no effect (the negligible differences between conditions are attributable to noise from the dimension selection procedure [33]); see Figure 5 (top). By contrast, when only world knowledge is manipulated (P(order(popcorn)|cinema)>P(order(dinner)|cinema)), and linguistic experience is held constant (“*NP${}_{person}$ entered the cinema and ordered popcorn/dinner*” are equally frequent), both online surprisal (S${}_{\mathrm{onl}}$) and online entropy reduction (ΔH${}_{\mathrm{onl}}$) pair with offline situation surprisal (S${}_{\mathrm{sit}}$) in that “dinner” is more effortful than “popcorn”, while offline linguistic surprisal (S${}_{\mathrm{ling}}$) shows no effect (Figure 5, middle). Finally, when there is a mismatch between linguistic experience (“*NP${}_{person}$ ordered champagne*” is more frequent than “*NP${}_{person}$ ordered water*”) and world knowledge (P(order(champage)<P(order(water))), online surprisal (S${}_{\mathrm{onl}}$) pairs with offline linguistic surprisal (S${}_{\mathrm{ling}}$) in that “water” is more effortful than “champagne” (Figure 5, bottom). Online entropy reduction (ΔH${}_{\mathrm{onl}}$), in turn, again aligns with offline situation surprisal (S${}_{\mathrm{sit}}$) in that “champagne” is more effortful than “water”. Indeed, a correlation analysis between online entropy reduction and offline situation surprisal for all words in the training data reveals a strong positive correlation between the two metrics (r=0.834, p<0.01).

In addition to comparing online entropy reduction (ΔH${}_{\mathrm{onl}}$) and online surprisal (S${}_{\mathrm{onl}}$) to offline linguistic surprisal (S${}_{\mathrm{ling}}$) and offline situation surprisal (S${}_{\mathrm{sit}}$), we could gain further insight by comparing them to the entropy reduction counterparts of these offline metrics: offline linguistic entropy reduction (ΔH${}_{\mathrm{ling}}$) and offline situation entropy reduction (ΔH${}_{\mathrm{sit}}$), which can both be straightforwardly estimated from the sentence-semantics pairs in the training data [45]. However, as all contrasts shown in Figure 5 concern sentence-final contrasts, there will in fact be no effects on linguistic entropy reduction: within each contrast, entropy will be the same for the control and target conditions at the penultimate word position, and in both conditions, the sentence-final words will reduce entropy to zero. Hence, there will be no difference in linguistic entropy reduction between the conditions. As for offline situation entropy reduction, this could be estimated by replacing ${\vec{v}}_{t}$ in Equation (6) with the situation vector $\mathrm{sit}(w_{1,\ldots ,t})$—the disjunction of all meaning vectors consistent with the prefix $w_{1},\ldots ,w_{t}$—that is also used for offline situation surprisal (see above). However, it turns out that for the model at hand, this yields the exact same predictions as offline situation surprisal (ΔH${}_{\mathrm{sit}}$) and hence will not lead to any further insights. This is a mathematical artefact of using strictly binary meaning vectors that represent disjunctions over propositions, which yield a uniform probability distribution over models. Under such a constellation, offline situation entropy and offline situation surprisal will not diverge, as the former is the weighted average of the latter.

In sum, online entropy reduction makes different predictions than online surprisal; while online surprisal reflects expectancy based on linguistic experience (∼offline linguistic surprisal) and world knowledge (∼offline situation surprisal), online entropy reduction consistently aligns with world knowledge and appears relatively insensitive to linguistic frequency differences.

### 3.3. Online Entropy Reduction as the Sentence Unfolds

**Figure 6:**
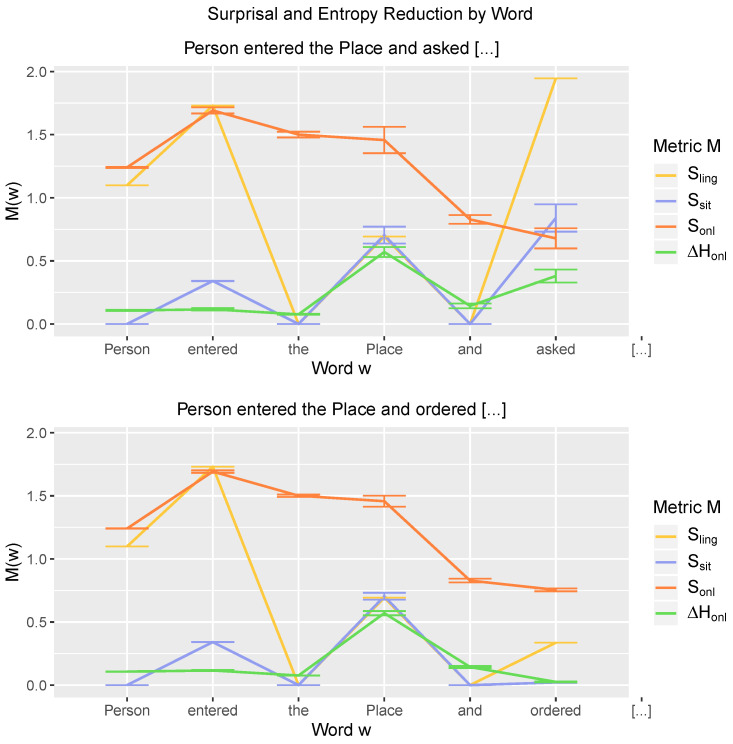
Word-by-word surprisal and entropy reduction metrics for two sets of sentences: “*NP${}_{person}$* entered the *NP${}_{place}$* and asked […]” (n = 6, top) and “*NP${}_{person}$* entered the *NP${}_{place}$* and ordered […]” (n = 30, bottom). Metrics shown are linguistic surprisal (S${}_{\mathrm{ling}}$), situation surprisal (S${}_{\mathrm{sit}}$), online surprisal (S${}_{\mathrm{onl}}$), and online entropy reduction (ΔH${}_{\mathrm{onl}}$). Error bars show standard errors.

Figure 6 shows the development of the surprisal and entropy reduction metrics across two sets of sentences “*NP${}_{person}$* entered the *NP${}_{place}$* and asked […]” (top) and “*NP${}_{person}$* entered the *NP${}_{place}$* and ordered […]” (bottom). These sets of sentences differ in terms of structural frequency (linguistic experience) and probability of their corresponding semantics (world knowledge): the latter sentences (ordering something after entering a place) are both more frequent and their semantics more probable than the former (asking for something after entering a place). This difference is reflected in the offline linguistic surprisal (S${}_{\mathrm{ling}}$) and offline situation surprisal (S${}_{\mathrm{sit}}$) metrics at the verb of the coordinated sentence, which is in non-final position: both predict higher surprisal for “asked” relative to “ordered”.

Indeed, online entropy reduction (ΔH${}_{\mathrm{onl}}$) aligns with the trajectory of offline situation surprisal (S${}_{\mathrm{sit}}$). Being consistent with both offline surprisal metrics, it predicts larger entropy reduction at “asked” than at “ordered” (ΔH${}_{\mathrm{onl}}\left(\mathrm{asked}\right)-\Delta$H${}_{\mathrm{onl}}\left(\mathrm{ordered}\right)=0.38-0.02=0.36$). By contrast, online surprisal (S${}_{\mathrm{onl}}$) follows a completely different path as the sentences unfold: after the second word, it does not align with any of the other metrics. Instead, online surprisal is relatively high at the beginning of the sentence, and at the critical words, it predicts only a slight (negative) difference in surprisal between “asked” and “ordered” ($S_{\mathrm{onl}}\left(\mathrm{asked}\right)-S_{\mathrm{onl}}\left(\mathrm{ordered}\right)=0.68-0.75=-0.07$). Hence, online entropy reduction and online surprisal develop differently as the sentences unfold, and they make qualitatively different predictions at the critical verb.

**Figure 7:**
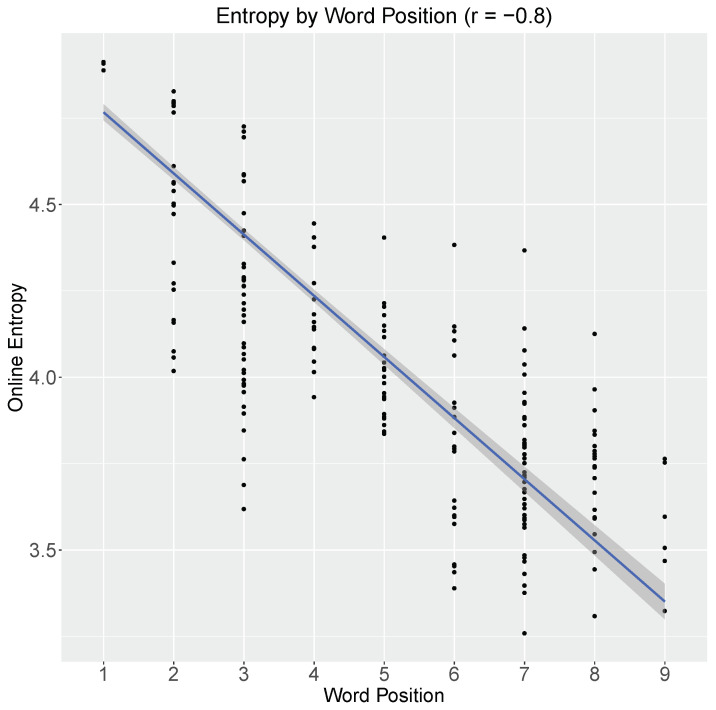
Online entropy by word position. The solid blue line depicts a linear regression and its 95% confidence interval. The Pearson correlation coefficient (*r*) is shown at the top.

The trajectory of online entropy reduction is relatively straightforward to understand: entropy reduction stays relatively low throughout the sentence, except for the points at which propositional meanings can be singled out (i.e., at “Place”, *enter(Person,Place)* is derived, and at “asked”, *ask_menu(Person)*). In turn, online surprisal is relatively high for the sentence-initial words. This is due to the way in which the model navigates through meaning space; it will start out with relatively uniform meaning vectors (∼high entropy, see Figure 7 below) and gradually move toward more polarized vectors with more units approximating 0 and 1 (∼lower entropy). Since surprisal derives from the conditional probability between two model-derived meaning vectors, it will be affected by the amount of polarization of these vectors, i.e., less polarized vectors generally lead to higher surprisal. Indeed, if we quantify the polarization at *t* in terms of entropy H(t) and the interaction in entropy between time step t−1 and *t* as H(t−1)*H(t), we obtain a significant positive relationship between surprisal and this interaction (r=0.532, p<0.01). In fact, this also explains the differential effect of surprisal and entropy reduction at the critical word: the vector ${\vec{v}}_{asked}$, constructed after processing “asked”, is more polarized than the vector ${\vec{v}}_{ordered}$, constructed after “ordered” ($|\{i|{\vec{v}}_{asked}\left(i\right)<0.1\vee {\vec{v}}_{asked}\left(i\right)>0.9\}|=126$ and $|\{i|{\vec{v}}_{ordered}\left(i\right)<0.1\vee {\vec{v}}_{ordered}\left(i\right)>0.9\}|=91$, for the sentences “thom entered the restaurant and asked/ordered”), since “asked” directly disambiguates the sentence-final meaning and “ordered” does not (“asked” is necessarily followed by “for the menu”, whereas “ordered” has different continuations, such as “cola”, “dinner”, etc.). As the amount of polarization affects the conditional probability, it may thereby obscure the effect of linguistic experience and world knowledge reflected in the two offline surprisal measures.

Since online entropy is defined relative to fully specified states of affairs, which are themselves represented as meaning vectors identifying unique models in M, entropy effectively quantifies the amount of polarization of the meaning vectors; low entropy states are more polarized. To illustrate how this polarization develops as the sentence unfolds, Figure 7 shows the entropy at each word of each training sentence of the VCB model (note that the distribution of sentence-lengths in the training data is as follows: 2 words (6), 3 words (150), 4 words (3), 5 words (18), 6 words (6), 7 words (33), 8 words (15), and 9 words (6); for a detailed description of the training data, see [33]). A first thing to note is that entropy reduces as sentences unfold (r=−0.8; p<0.01). As the model processes sentences on a word-by-word basis, it moves through points in space that render it increasingly clear which propositions are the case and which are not, thereby reducing uncertainty about the state of affairs conveyed by the utterance. Secondly, this figure shows that sentence-final entropy remains relatively high (in comparison to the maximum entropy for the 150 non-duplicate models constituting the meaning space in [33], which is: $-\log(\frac{1}{150})=5.01$ nats (=7.23 bits)). Indeed, when entropy is defined relative to fully specified states of the world, individual sentences will not reduce entropy to zero (in contrast to previous instantiations of linguistic entropy that are defined over sentence-final structures [4,8,14,15]): for instance, the sentence “beth ordered cola” is satisfied by all models in which *order(beth,cola)* is the case but is not explicit about all the other propositions that can co-occur with it, thus leaving significant uncertainty with respect to the fully-specified state of affairs.

## 4. Discussion

We have derived a comprehension-centric notion of online semantic entropy, based on a comprehension model that incrementally constructs probabilistic distributed meaning representations. Instead of defining entropy over the probabilistic structure of the language, we here define it in terms of the structure of the world [45]. That is, in line with the comprehension-centric notion of surprisal presented by VCB [33], entropy derives from the model’s incremental navigation through meaning space, which is guided by both linguistic experience and world knowledge [33]. More specifically, at time step *t*, entropy in this model quantifies the amount of uncertainty at *t* with respect to fully specified states of affairs, i.e., the combinations of propositions that constitute the meaning space.

While surprisal is estimated from the probabilistic properties of previous and current states of processing—and hence naturally falls out of probabilistic language (processing) models—entropy derives from the probabilities of all possible future states (e.g., every possible continuation of the sentence at hand), which makes it typically less straightforward to estimate. Indeed, given that the set of possible sentences that can be produced is non-finite, this quickly becomes infeasible, and some state-limiting mechanism is required in order for entropy to be estimated (e.g., see [15]). In the present model, by contrast, this is mitigated by the fact that entropy, like surprisal, directly derives from the finite dimensions of the utterance meaning representations that the model constructs on a word-by-word basis. That is, at each time step *t*, the model produces a vector $\vec{v}\left(t\right)$ representing the activity pattern over |M| neuron-like processing units, and entropy directly derives from these |M| states. While this offers an account of entropy (and surprisal) at the level of representations—and hence at Marr’s [16] representational and algorithmic level—it does raise questions about the ecological status of M. We see M as a set of representative, maximally informative models reflecting the structure of the world. That is, we do not take each M∈M to instantiate a single observation of a state-of-affairs but rather as an exemplar state-of-affairs, which combines with the other exemplars in M to represent the probabilistic structure of the world. In this sense, M can be seen as an abstraction of our accumulated experience with the world around us. Indeed, this gives rise to the question of how M could be acquired, developed, and altered as children and adults navigate the world over time. While this is a question for language acquisition that is beyond the scope of this article, one speculative approach could be to implement M as a self-organization map (SOM), which consists of the running average of maximally informative states of affairs (e.g., see [37]) and which interfaces with the comprehension model. Of course, despite this perspective on the set of states of affairs M that constitutes our meaning space, the number of dimensions needed to capture real human world knowledge will significantly exceed the limited dimensions of the current model. As a result, entropy is predicted to be high in general, and individual sentences are predicted to reduce entropy only marginally. Critically, however, sentences are generally interpreted in context (be it a linguistic or extra-linguistic context), which significantly constrains the set of states of affairs that contribute to the word-derived entropy: for instance, a context in which “beth enters the restaurant” will effectively reduce our meaning space to only those states of affairs that are related to (beth) going to a restaurant. Hence, entropy calculation regarding fully specified states of affairs becomes both feasible and intuitive when taking a context-dependent (or dynamic) perspective on language comprehension.

Using the comprehension model presented in [33], we have investigated how the comprehension-centric notion of entropy reduction behaves during online comprehension and how it relates to online surprisal. We have found that online entropy reduction and surprisal correspond to differential processing metrics, which may be reflected in different behavioral effects (cf. [15]). Critically, entropy reduction and surprisal here are not conceived as reflecting different underlying cognitive processes as both derive from the model’s comprehension process as navigation through meaning space. They do, however, describe distinct aspects of this navigation process; whereas surprisal reflects the transition in meaning space from one word to the next, entropy reduction quantifies how much uncertainty is reduced with respect to the state of the world. This explains why entropy reduction seems less sensitive to effects of linguistic experience than surprisal; even though the point in meaning space at which the model arrives at time step *t* is determined by both linguistic experience and world knowledge (as reflected in the online surprisal estimates [33]), entropy is calculated relative to fully specified states of affairs, which means that it will be more sensitive to probabilities that derive from the structure of the world than to those deriving from linguistic frequency effects. This is especially true in the current setup of the model, where linguistic experience is limited to word frequency effects (sentence structures are relatively invariant across the training data). Hence, to the extent that linguistic experience can restrict which states of affairs are consistent with the current meaning vector, it may affect online entropy reduction. However, the presented set of contrasts illustrates that online surprisal is inherently more sensitive than entropy reduction to effects of linguistic experience. Overall, the observation that entropy reduction is highly sensitive to the probabilistic structure of the world is consistent with recent findings from situated language comprehension [34].

A consequence of deriving entropy from fully specified states of affairs is that entropy stays relatively high after processing sentence-final words. As discussed above, this is because of the structure of the world and the world knowledge-driven inferences that are inherent to the meaning representations: after a sentence is processed, its literal propositional content and any highly likely or necessary propositions that co-occur with it, are inferred to be the case, but there also remains a vast amount of uncertainty regarding other propositions that could co-occur with it. This is consistent with a perspective on language comprehension in which pragmatic inference is an inherent part of incremental, word-by-word processing. In fact, one could argue that the model instantiates a perspective in which comprehension *is* pragmatic inference; the literal propositional content of an utterance has no special status—there is only the probabilistic inferences that derive from processing an utterance (which will typically entail the literal propositional content). This leads to another prediction regarding the difference between surprisal and entropy reduction in our model: surprisal, which derives directly from two subsequent points in meaning space, effectively reflects how the likelihood of inferred propositions changes *locally*, as it only takes into account the inferences contained within these points. Entropy reduction, in turn, looks at the difference in entropy between these points, which explicitly factors in the likelihood of all possible inferences. Entropy reduction thus reflects how the likelihood of inferred propositions changes *globally*, i.e., with respect to the full set of possible inferences that could be drawn. Hence, in the current instantiation of the model, the surprisal of the word “restaurant” in the sentence “beth entered the restaurant” is driven by the change in likelihood between the (probabilistic) inferences made at the word “the” and those made at the word “restaurant”, while its entropy reduction is determined by the difference in uncertainty about the full set of inferences available to the model.

In sum, in the comprehension-centric perspective on surprisal and entropy reduction formalized in the current model, the metrics derive from a single process—word-by-word meaning space navigation—but differ in which aspects of this process they elucidate. That is, the processing of an incoming word moves the model from a previous point to a next point in space. The exact coordinates of these points depend on the linguistic experience of the model as well as the world knowledge contained within the meaning space that it navigates. Surprisal quantifies how likely the next point is given the previous one and thereby effectively how expected the input was. Surprisal can thus be thought of as reflecting *state-by-state expectation*, where input that moves the model to unexpected points in space yields high surprisal. Entropy, in turn, quantifies how likely each fully-specified state of affairs constituting the meaning space is, given the current point in space. Entropy reduction, then, is effectively a metric of *end-state confirmation*, where higher reduction of uncertainty about the propositions that are communicated to be the case, i.e., stronger confirmation of the communicated state-of-affairs, leads to higher reduction of entropy. This characterization appears to be in line with recent theories and models from the text comprehension literature, in which the notion of *validation*—the process of evaluating consistency of incoming linguistic information with the previous linguistic context and general knowledge about the world—has a central role [46,47,48]. The above described conceptualization of entropy reduction in terms of end-state confirmation might indeed turn out to be an index of the degree of, or effort induced by, validating the incoming input against the larger context and knowledge about the world. To the extent that this mapping is correct, one could explore the dissociation between entropy reduction and surprisal even further by turning to experimental designs that pit global knowledge of the world against local textual/discourse coherence—a good point to start this investigation is by turning to the text comprehension literature [17,19,21,27,49,50].

Taken together, the conceptualization of comprehension as meaning-space navigation predicts a dichotomy in which surprisal and entropy reduction—while often correlated—differentially index effort during incremental, expectation-based comprehension: state-by-state expectation (surprisal) versus end-state confirmation (entropy reduction). That is, while both metrics derive from transitions between states in meaning space, surprisal approximates the distance of this transition, whereas entropy reduction reflects a change in the inherent nature of these states: the degree of certainty regarding the state of affairs being communicated.
